# Early development and coloniality in *Oligophylloides* from the Devonian of Morocco—Are Heterocorallia Palaeozoic octocorals?

**DOI:** 10.1371/journal.pone.0257523

**Published:** 2021-09-29

**Authors:** Błażej Berkowski, Mikołaj K. Zapalski, Emilia Jarochowska, Phil Alderslade

**Affiliations:** 1 Institute of Geology, Adam Mickiewicz University, Poznań, Poland; 2 Faculty of Geology, University of Warsaw, Warsaw, Poland; 3 GeoZentrum Nordbayern, Friedrich-Alexander-Universität Erlangen-Nürnberg, Erlangen, Germany; 4 Division of Oceans & Atmosphere, The Commonwealth Scientific and Industrial Research Organisation (CSIRO), Hobart, Tasmania, Australia; Naturhistoriska riksmuseet, SWEDEN

## Abstract

Heterocorals represent an enigmatic group of Palaeozoic corals, known from relatively short time intervals in the Devonian and Carboniferous periods. The major differences between Heterocorallia and other Palaeozoic corals are the lack of an external theca (epitheca), lack of calices and the presence of dichotomously dividing septa-like structures. Heterocoral skeleton was presumably externally covered by the soft tissue and each branch of their skeleton has, until now, been regarded as a corallite–a skeleton of a single polyp. We investigated upper Famennian *Oligophylloides* from Morocco, focussing on branching processes, wall structure, previously poorly known initial growth stages and the growing tip, described here for the first time. We demonstrate that *Oligophylloides* shows a unique colony development not known in any group of anthozoans possessing a septate-like architecture and suggest that the previously postulated homology between true septa in hexa- and rugose corals on one hand, and *Oligophylloides* on the other, must be rejected. Based on the skeleton structure and branching patterns, we postulate, contrary to former ideas, that the stem and branches of heterocorals represent the skeleton of a multi-polyp colonial coral, similar to many extant octocorals. We found numerous potential homologies with octocoral skeletons (notably the Keratoisidinae within the Isididae) and, as a result, we propose the inclusion of the order Heterocorallia within the subclass Octocorallia. This suggestion requires, however, further research on the other taxa of heterocorals. We also propose some changes to the morphological terminology for the Heterocorallia.

## Introduction

The Devonian *Oligophylloides* Różkowska, 1969 since its erection [1] became one of the most studied genera of the order Heterocorallia Schindewolf, 1941. Nevertheless, heterocorals [2] still remain a problematic extinct group, with unclear relationship to other Palaeozoic and extant cnidarians. The main characters distinguishing the skeletons of heterocorals from the skeletons of contemporary Palaeozoic rugose and tabulate corals is that they did not develop an external theca (epitheca) nor calices [e.g. 3–7]. Another distinguishing character is what has been referred to as the septal apparatus that is composed of dichotomously dividing septa, which formed centrifugally in the distal cone and in the axial part of the branch [see detailed discussion in 4]. These features clearly show that the calcite skeletons of heterocorals must have been covered externally by soft tissue, which produced a more or less thick internal heterotheca [see 5,6]. Two views concerning the origin of heterocorals have recently been presented: (1) that heterocorals derived from rugose corals [4,8] or (2) that they evolved from other, non-skeletonised cnidarians [see discussion in 9].

The order Heterocorallia is relatively poorly represented in the fossil record and contains only six genera occurring in the Devonian and Carboniferous periods. The oldest and rarest records of heterocorals are known from the Eifelian Stage [10,11], but they became relatively common over short time intervals during the late Famennian and the Visean ages. The last representatives are recorded from the upper Serpukhovian [12]. There are, however, long-term gaps in their occurrences and, surprisingly, Heterocorallia are unknown from the Givetian, Frasnian and the upper Tournaisian. In the Famennian, they are represented by two genera: *Oligophylloides* Różkowska, 1969, which is more common, and *Mariaephyllia* Fedorowski, 1991. Both taxa inhabited relatively deep-water (mesophotic and aphotic), apparently oxygen-depleted environments of the late Famennian seas [1,5,6,9,13] and co-occurred with solitary undissepimented rugose corals—the so-called *Cyathaxonia* fauna *sensu* Hill (1938) [14] and rare tabulates [15].

In the fossil record, complete coralla of heterocorals are extremely rare [6] due to the fragile architecture of their skeletons composed of long and relatively thin branches. Hence, in most cases they are found as isolated broken branches lacking their proximal and distal parts. The most complete and relatively large colonies of *Oligophylloides* have been figured by Weyer [6]. The striking character of the colony morphology is that the branches, if present, were developing in one plane, forming fan-shaped coralla. Although the distal parts of coralla have not been found up to now, their suggested morphology was reconstructed for the genus *Oligophylloides* and interpreted as narrowing distal cones of individual corallites [7,16]. On the other hand, the proximal parts have been already documented in several specimens from the Famennian *Oligophylloides* from the Holy Cross Mts. (Poland) [1,3] and interpreted as talons (extending attachment structures) where laminae of heterotheca are partly separated from each other and central lumen with a developed septal apparatus. However, the material investigated here shows that the specimens studied by these authors were incomplete and hence the early ontogeny was probably misinterpreted. It seems that, among all previously studied proximal parts, the most complete was the one figured by Weyer (1997, his Fig 2) [5], in which an aseptate hollow is present in the central part during the initial stages of development. This peculiar type of early development of the skeleton was interpreted by him as anomalous and unique, but the material described here shows that such mode of development is typical and common in *Oligophylloides*. Furthermore, the basal part attached to the substrate represents a structure and origin that is different from that of a ’true’ talon developed in rugose corals, for which the term ’attachment body’ was proposed [4]. More massive, proximal parts of *Oligophylloides* coralla, composed of many individual branches united within a common thick protoheterotheca (paracolonies *sensu* Weyer 2017), have been described from the Famennian of Morocco [5,6,17].

In the present study, we describe the earliest growth stages and branching process in the genus *Oligophylloides* from the Famennian of Morocco, based on exceptionally well preserved juvenile specimens and branches of mature parts of the coralla. Additionally, one studied juvenile specimen possesses a perfectly preserved distal part, figured here for the first time. Our detailed analyses of external and internal structures of these juvenile coralla show a unique, atypical development of their skeleton, unknown in any other fossil coral groups. However, the material displays numerous characters closely resembling typical traits of extant octocorals and we, therefore, suggest a revision of the systematic position of Heterocorallia.

## Terminology

Due to the new concept of the anatomy and systematic affinities of Heterocorallia presented here, we present a combination of established heterocoral and octocoral terminology to describe the known and previously unknown parts of the skeleton:

**Axis** (new term for Heterocorallia)–the inner supporting skeletal structure.

**Branch** (new term for Heterocorallia)–a lateral growth of the corallum of heterocorals, composed of a central core with a system of parasepta and tabulae surrounded by heterothecal and/or protoheterothecal layers–formerly referred to as a corallite.

**Central core** (new term for Heterocorallia)–the hollow central channel of the colonial axis where the system of parasepta and tabulae (formerly known as the tabularium) are formed, surrounded by heterothecal layers.

**Growing tip** (new term for Heterocorallia)–the distal part of a colonial stem or branch with protruding paraseptal apparatus, characterised by a wall of the heterothecal type (see below)–formerly referred to as the distal cone (*sensu* Wrzołek 1993 [16])

**Heterotheca** (after Schindewolf 1941 [2]—modified)–usually the inner, mostly lamellar wall surrounding the hollow central core and internally composed of paraseptal and interparaseptal sectors, respectively. If exposed as an outer wall, external longitudinal ribs and furrows may be present.

**Holdfast** (new term for Heterocorallia)–circular, ellipsoidal or irregular extended proximal part of the stem attaching the corallum to the substrate and consisting of a hollow central core surrounded by a protoheterotheca with partly separated laminae. Previously referred to in Heterocorallia as: 1) talon by Różkowska 1996 [1]; 2) attachment body by Fedorowski 1991 [4].

**Parasepta** (new term for Heterocorallia)–septal-like structures developed in the central core of the axis, formed by peripheral dichotomous division of the first axially located paraseptum (oblique septum *sensu* Fedorowski 1991 [4]). Interpreted here as not secreted in the mesenteries of a single polyp and therefore not analogous functionally to true septa of corals.

**Protoheterotheca** (after Fedorowski 1991 [4]—modified)–the fully lamellar smooth external part of the wall, lacking traces of incorporated parasepta.

**Stem** (new term for Heterocorallia)–the unbranched part of the corallum arising from the holdfast or the basal part of a branched corallum arising from the holdfast.

## Geological setting

**Fig 1:**
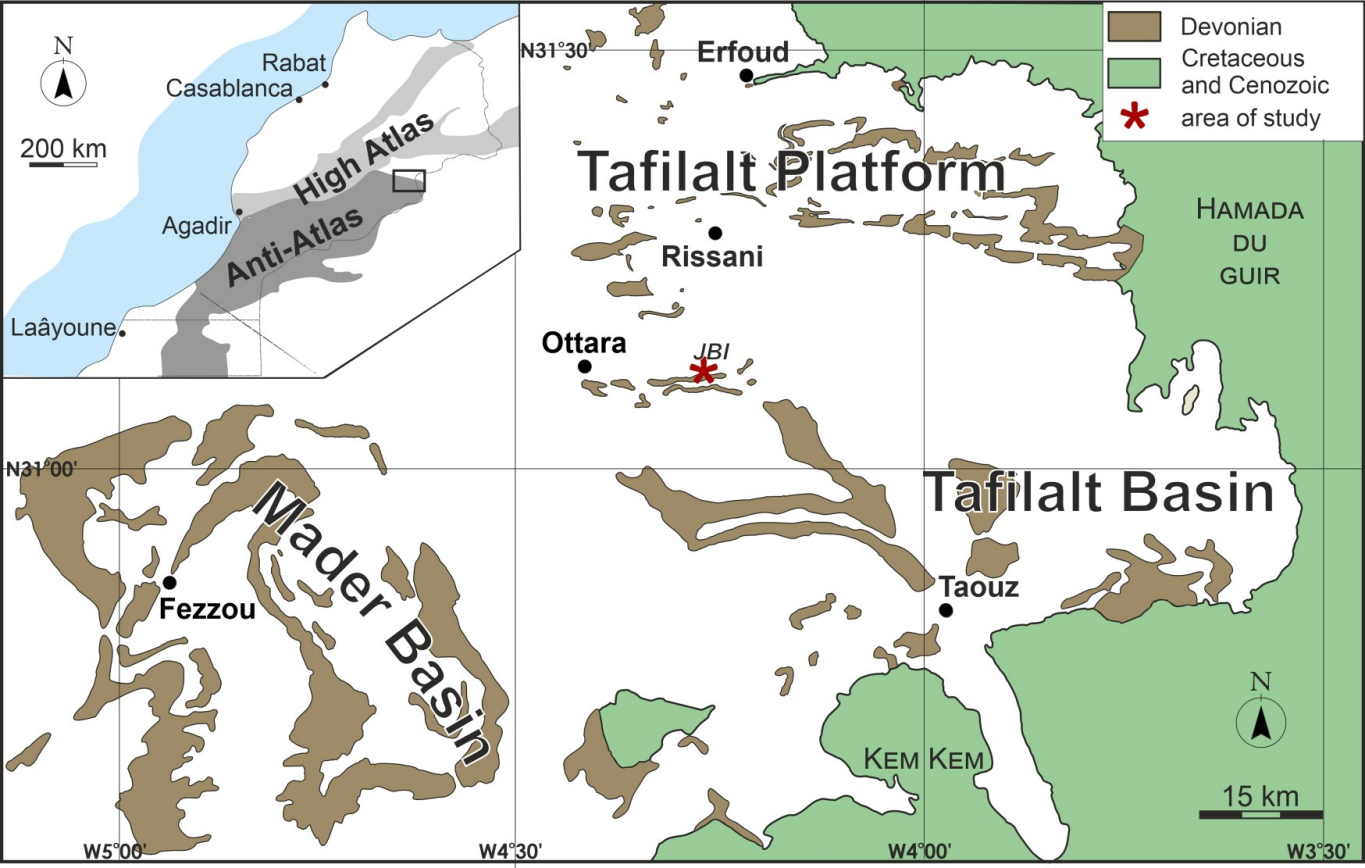
Simplified geological map of the eastern Anti-Atlas with indicated Devonian outcrops. Republished from [18] under a CC BY license, with permission from American Geophysical Union, original copyright 2009. Slightly modified, the location of Jebel Bou Ifarherioun is indicated by a star. Inset shows a general geological context of the study area.

The studied material was collected in the Jebel Bou Ifarherioun ridge, located approximately 16 km SSW of Rissani (eastern Anti‐Atlas, Morocco; Fig 1).

The ridge extends longitudinally about 5 km from the east to the west and represents the elevated northern limb of the syncline composed of the Middle and Upper Devonian carbonate rocks [19]. In the Devonian Period, this area was located on the shelf developed upon the north‐western passive margin of Gondwana, forming the Tafilalt Platform. The studied specimens of *Oligophylloides* were found in the trenches exploited for their ammonoid fossils and limestone blocks. These trenches are located on the southern slope of the Jebel Bou Ifaheriuon ridge in its western part (coordinates: 31°07’57.3"N 4°18’28.8"W). The carbonate beds cropping out in the trenches represent the upper Famennian and contain grey‐to‐reddish crinoid wackestones/packstones enriched in ammonoids, mainly clymeniids, goniatids and orthocone nautiloids. Of the ammonoids, the most characteristic are the specimens of *Gonioclymenia speciosa* [see e.g. 20], hence the horizon is known in the literature as the *Gonioclymenia* Limestone [21]. Corals are relatively numerous and dominated by heterocorals (*Oligophylloides* and *Mariaephyllia*), extremely diverse solitary undissepimented rugosans and rare tabulate coralla are also present (currently under study). Associated biota also include numerous reworked and broken crinoid remains, trilobites, bryozoans, brachiopods, bivalves, gastropods and fish fragments. The *Gonioclymenia* Limestone was dated to the upper Famennian—middle/upper *Palmatolepis gracilis expansa* conodont Zone *sensu* [22], which corresponds to the Subzone of *Bispathodus costatus* (within *Bispathodus aculeatus aculeatus* Zone) according to the zonation proposed in [23] and [21].

## Material and methods

The studied material belongs to the genus *Oligophylloides* Różkowska, 1969, and was collected from the *Gonioclymenia* Limestone (upper Famennian) cropping out in the western part of Jebel Bou Ifarherioun during fieldwork campaigns in 2019 and 2020. The studied fossils comprise 26 small specimens (*c*. 15–36 mm long), representing juvenile (proximal) parts, and more than 30 broken, fragmentary branched sections representing more distal parts of the coralla. One specimen comprises the upper part of a fan-shaped colony with seven branching points and one point where two neighbouring branches anastomose. Each of the studied juvenile specimens possesses a well-preserved holdfast. Four juvenile specimens are encrusted by small colonies of tabulates (auloporids and pachyporids), one by a solitary rugose coral and one by the heterocoral *Mariaephyllia*. Some of these epibionts are partly incorporated in the heterocoral skeleton. The specimens were collected under the collection permit (Attestation) issued by Directeur de la Direction de la Géologie Dr. Ahmed Benlakhdim at Ministère de l’Energie, des Mines, et de l’Environnement (Rabat, Morocco), for Błażej Berkowski.

To analyse the internal structure of the most juvenile (proximal) parts, two specimens were ground down and polished to obtain serial transverse acetate peels and one specimen was sliced to obtain 16 thin, serial, transverse sections. Two juvenile specimens were cut to make thin longitudinal sections through their hollow central core. Four specimens representing the upper parts of branched coralla were cut to prepare thin longitudinal sections. All sections were made using a Unipress WS-20 wire saw with a 0.05 mm wire diameter. Additionally, three specimens were analysed using a Phoenix v|tome|xs research edition computer tomographic scanner at GeoZentrum Nordbayern (Erlangen, Germany). Specimens and thin sections were investigated and photographed using a Zeiss Discovery V20 Stereomicroscope equipped with a Canon 70D camera. One specimen with a well-preserved growing tip was photographed using a Hitachi 3700N Scanning Electron Microscope. All of the studied specimens are housed at the Adam Mickiewicz University, Poznań, Poland (Institute of Geology) under collection numbers UAM/B/He/XXX. Underwater images of octocoral colonies were taken by towed camera on various seamounts south of Tasmania, Australia. The following isidid specimens used to investigate axis structure are held at The Commonwealth Scientific and Industrial Research Organisation (CSIRO) Division of Oceans & Atmosphere, Hobart, Tasmania: *Isidella* sp. a, IN2018-V06-17-18, south of Tasmania, -44.267°, 147.109°, 1139 m, 25 Nov. 2018, RV *Investigator*; *Acanella* sp. d, IN2018-V06-184-13, north east of Tasmania, -41.204°, 148.789°, 1127 m, 17 Dec. 2018, RV *Investigator*; *Primnoisis* sp. n, IN2018-V06-94-33, south of Tasmania, -41.106°, 146.203°, 930 m, 6 Dec. 2018, RV *Investigator*; *Keratoisis* sp. k, IN2018-V06-105-4, south of Tasmania, -44.096°, 146.679°, 568 m, 8 Dec. 2018, RV *Investigator; Keratoisis* sp. a, SS200702 050–002, south of Tasmania, -44.206°, 146.197°, 1200 m, 6 April 2007, RV *Southern Surveyor*: *Isidella* sp. b, SS199701 47, south of Tasmania, -44.33°, 147.11°, 1200 m, 21 Jan. 1997, RV *Southern Surveyor*; *Jasonisis thresheri*, holotype, Tasmanian Museum & Art Gallery K3879, Tasman fracture Zone, south east of Tasmania, sample J2-391-020-001, -45.37°, 144.61°, 2063 m, ROV *Jason* deployed from the U.S. RV *Thomas T*. *Thompson*, Dr Jess Adkins & Dr Ron Thresher, 9 January 2009. All contemporary specimens used in this study were loaned from existing collections, and therefore no collection permits were required.

Image stacks and animations created from X-ray microtomographic reconstructions are available under http://morphobank.org/permalink/?P3954.

## Taxonomic remarks concerning the genus *Oligophylloides* Różkowska, 1969

The main character distinguishing the genus *Oligophylloides* from other genera of Heterocorallia is the very thick wall surrounding a central core within which there exists a kind of ’septal’ apparatus (here termed parasepta) and tabulae. In heterocorals, the wall was originally termed the ’heterotheca’ [2] and is present in most genera of this group. Subsequently, the most external part of the wall in *Oligophylloides* was termed the ’protoheterotheca’ [4]. The typical inner heterotheca differs from the outer protoheterotheca by including the continuations of the paraseptal insertions. To date, this kind of the wall composed of two structurally different layers has been reported only in representatives of this genus.

The taxonomy of the genus *Oligophylloides* is problematic due to the lack of ontogenetically stable skeletal characters, making determination of individual species difficult. Initially, three taxa were erected [1]: the type species *Oligophylloides pachythecus* Różkowska, 1969 and two additional subspecies: *Oligophylloides pachythecus pentagonus* Różkowska, 1969 and *Oligophylloides pachythecus tenuicinctus* Różkowska, 1969. The latter two taxa were synonymized with the type species, based on statistical measurements of the thickness of wall and diameter of central core in a large population from the type locality [3]. Further investigations [6,13] supported this view that the diameter and the thickness of the heterotheca are not sufficient to distinguish individual species, as they may vary within one specimen markedly. This is also the case for other quantitative characters, such as the diameter of the tabularium (here termed the central core) and the number of parasepta, which may change their arrangement or temporarily disappear in later growth stages. *Oligophylloides parvulus* Weyer, 1995 and *Oligophylloides weyeri* Berkowski, 2002 seem to also belong to the type species, with the type specimens most likely representing the most distal fragments (growing tips) of branches, as they developed a very thick wall of the heterotheca type, i.e. completely crossed by parasepta with external, longitudinal ribs [see also discussions in 6 and here]. The recently described species *Oligophylloides maroccanus* Weyer, 2017 is also problematic, as it forms large colonies with extremely thick-walled branches with a relatively narrow central core. These characters, however, seem to be rather an effect of long-time growth of the colonies of these Moroccan populations, perhaps resulting from favourable conditions. Consequently, the specimens described as *Oligophylloides maroccanus* Weyer, 2017 may also represent the type species.

For the purpose of this study the investigated specimens have been assigned only to the genus level, as most of them represent very young stages of growth that could have developed into forms recognisable as different species. Although they co-occur in the type locality and strata with *Oligophylloides maroccanus* Weyer, 2017, and would appear to be a part of the population of this peculiar Moroccan species, they may also belong to the type species if the new concept presented in this paper holds.

## Early development of *Oligophylloides*

### External characters

**Fig 2:**
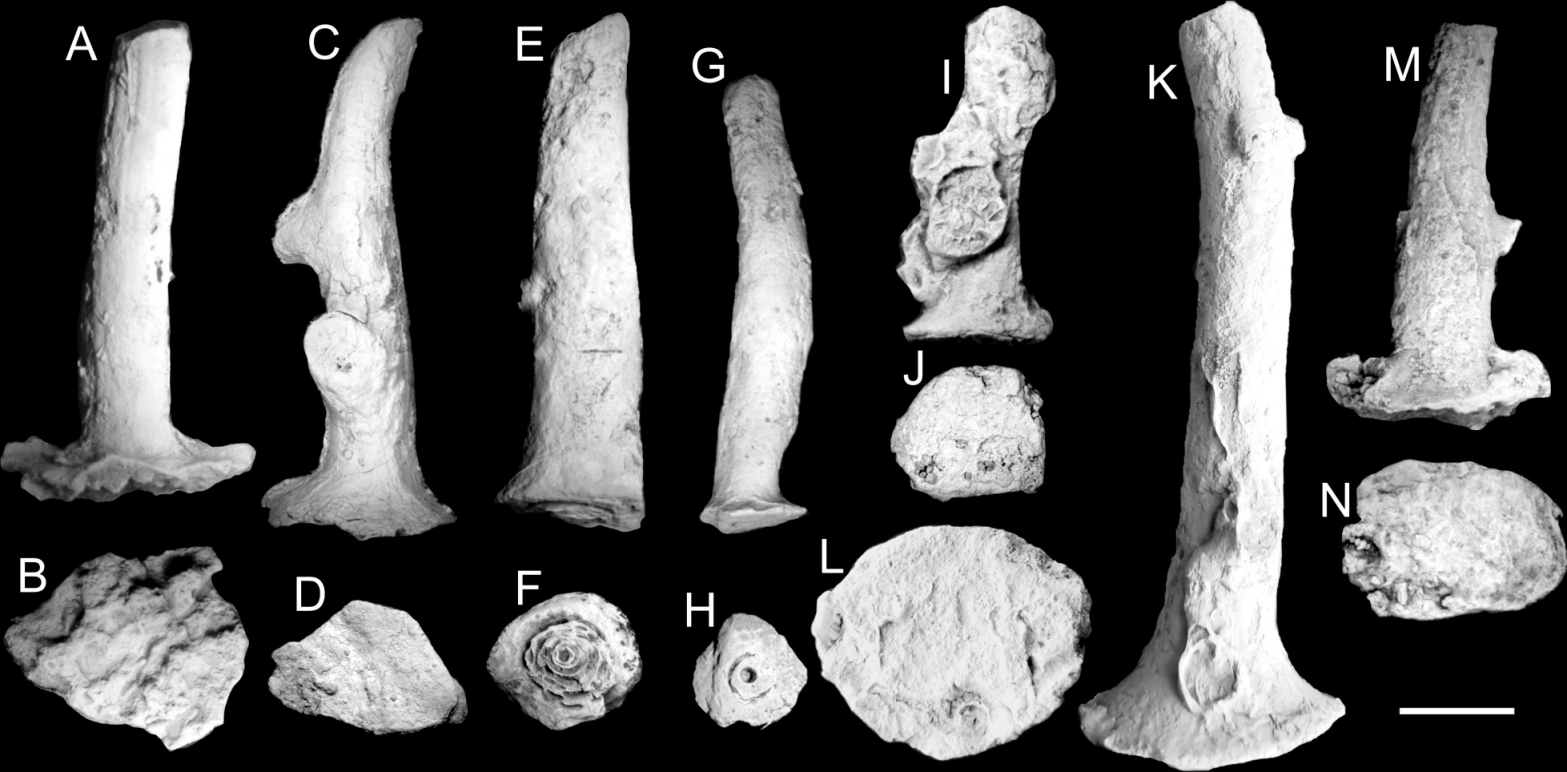
External view of studied juvenile specimens of *Oligophylloides* sp. (side and bottom view ordered respectively). A, B–specimen UAM/He/B/011; C, D–specimen UAM/He/B/012; E, F–UAM/He/B/013; G, H–specimen UAM/He/B/014; I, J–specimen UAM/He/B/015; K, L–specimen UAM/He/B/016; M, N–UAM/He/B/21. Detailed explanations in the text. Scale bar 5 mm.

**Fig 3:**
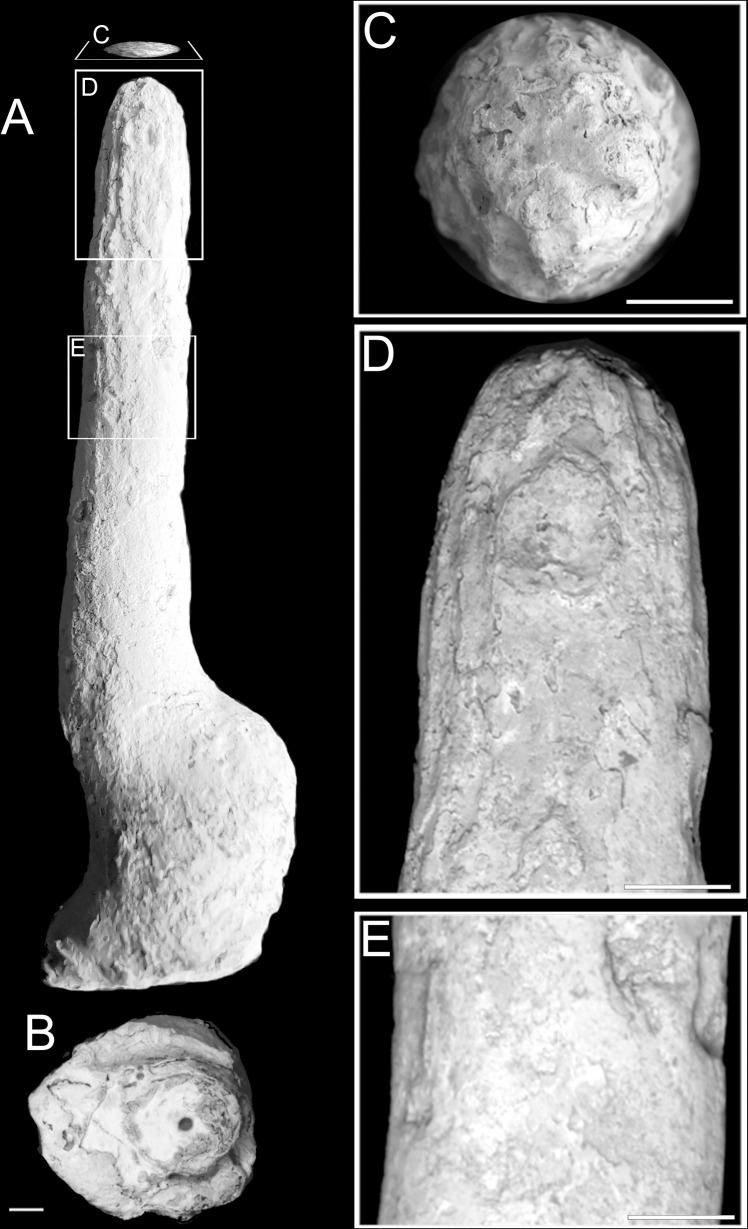
*Oligophylloides* sp.—Juvenile specimen UAM/He/B/022 with well-preserved growing tip. A, B–external view (side and bottom respectively). Scale bar 2 mm. C–SEM of apical part (top view) of the growing tip; D–SEM of side view of the growing tip with ribs (parasepta) and furrows (interparaseptal) sectors (heterotheca); E–SEM of side view of the smooth protoheterotheca. Scale bars for C, D, E 1 mm.

All studied specimens representing the ontogenetically youngest (proximal parts) of the skeleton possess a well-developed holdfast (Fig 2), but, apart from one specimen (Fig 3), they lack their most distal parts. Two specimens additionally developed branches after the initial growth, clearly showing that the branching process could start relatively early in the colony development (Figs 2C and 6).

**Fig 4:**
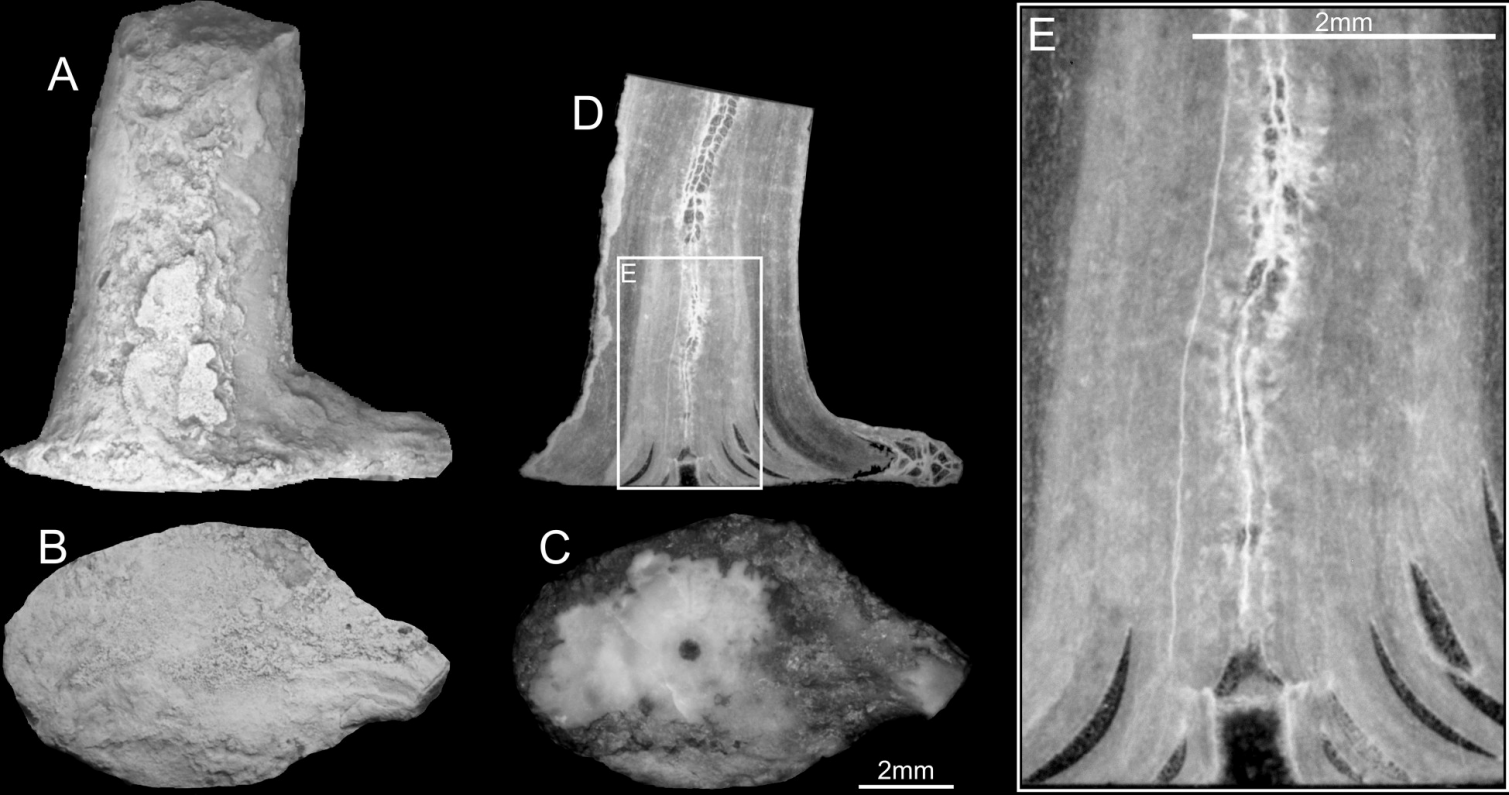
*Oligophylloides* sp.—Juvenile specimen UAM/He/B/017. A, B–external view (side and bottom); C–partly ground bottom of the attachment body (note the empty hole in the centre); D–longitudinal thin section of the specimen trough the central lumen of the protobranch; E–enlarged fragment of the lower part of the longitudinal section (D). Scale bar 2 mm.

**Fig 5:**
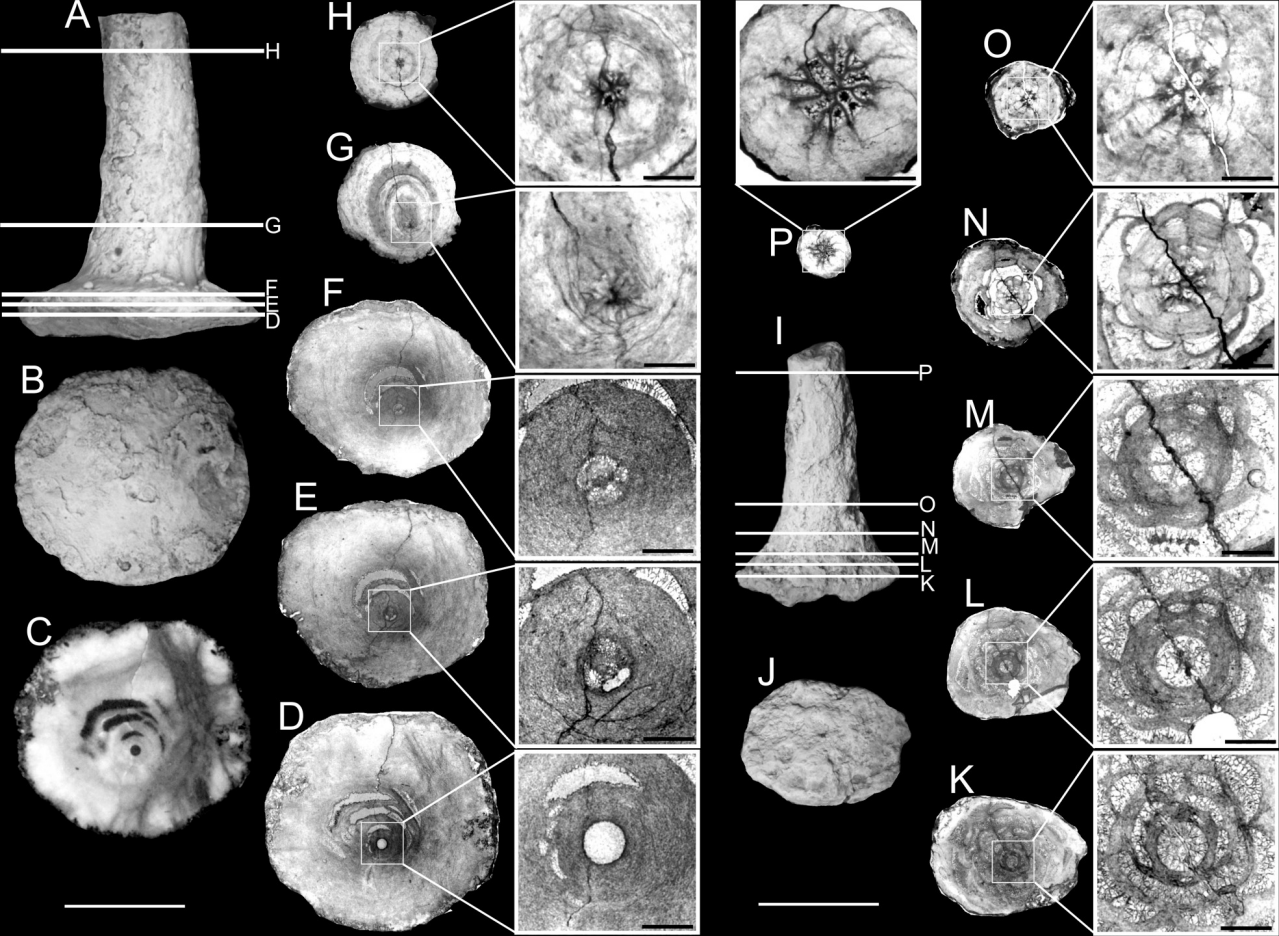
Two juvenile specimens of *Oligophylloides* sp.: A-H–UAM/He/B/018, I-P–UAM/He/B/019. A, B–external views (side and bottom); C–partly ground bottom part of the specimen (note empty hole in the centre and protoheterotheca with separated laminae of the attachment body); D-H–successive serial transversal sections (acetate peels and thin sections) showing successive ontogenetic growth stages. Enlarged parts of the sections indicating changes in the central lumen and surrounding protoheterotheca in squares. I,J–external views (side and bottom); K-P–D-H–serial transversal sections (acetate peels and thin sections) showing successive ontogenetic growth stages. Enlarged parts of the sections indicating changes in the central lumen and surrounding protoheterotheca in squares. Scale bar 2 mm, scale bars in the enlarged parts 1 mm.

The dimensions of juvenile stems vary: their length is *c*. 10–35 mm and the widest (proximal) part of the attachment body may exceed 10 mm in some well-developed specimens (Figs 2B, 2L, 2N and 5B). The shape of the bottom surface of the holdfast in the best-preserved specimens is circular, ellipsoid or irregular, depending on the shape and character of the substrate (Figs 2–5). The texture can be almost smooth (Figs 2D, 2J and 5B) or with numerous protrusions, often incorporating detrital elements of the substrate (Fig 2B and 2N). Three coralla have partly weathered proximal parts showing a centrally placed circular hollow with a diameter of 0.4–0.7 mm (Figs 2F, 2H and 3B). This circular hollow, filled with sediment, is also present at the base of the skeleton of all ground or cut specimens (Figs 4C and 5C). The external surface of the skeleton (protoheterotheca-type) is mostly smooth, but in several juvenile specimens wide longitudinal ribs are developed on the upper portion of the holdfast (see e.g. Fig 4A). Additionally, on the growing tip found in one well-preserved juvenile specimen (Fig 3A and 3D), the wall is of the heterotheca type with ribs and furrows, which may represent traces of protruding parasepta and depressed interseptal sectors within the wall.

The most apical part of the growing tip shows dichotomously dividing parasepta with an axial ’oblique’ paraseptum in the centre (Fig 3C). Detailed observations of apical parts in thin sections of other specimens show that divided parasepta may be also present within the heterotheca (Fig 5P).

### Internal characters—Longitudinal section

One specimen was cut longitudinally following the middle of the central core (Fig 4). For accurate sectioning the holdfast was partly ground to reach the initial hollow (Fig 4C). The section shows that the basal tabula is transversal and elevated 0.8 mm above the lowest laminae of the protoheterotheca, forming a cylindrical space filled with sediment (Fig 4D and 4E).

The parasepta began to be formed in the central core about 0.5 mm above the basal tabula, on the second tabula, which is strongly convex (Fig 4D and 4E). From here, the axis consists of heterotheca and protoheterotheca surrounding the central core forming successive laminae, which were secreted centrifugally. At the base, the protoheterotheca is composed of thick sets of laminae, which are in places separated, forming a basally extended holdfast, much wider and thicker than the upper part of the corallum.

### Internal characters—Transverse sections

**Fig 6:**
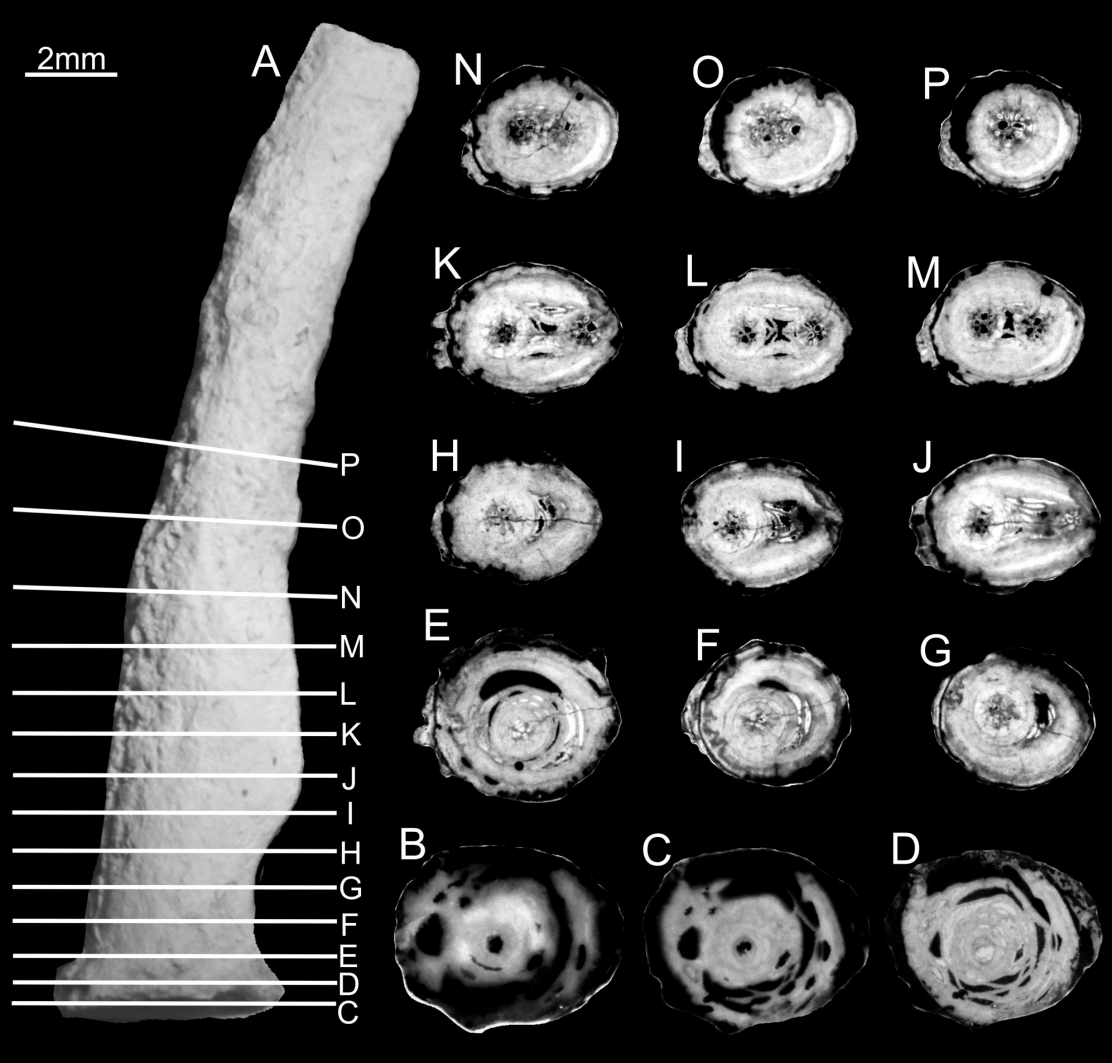
Juvenile specimen of *Oligophylloides* sp. UAM/He/B/020. A–external view, white lines show the planes of cuttings; B-P–successive serial transversal thin sections showing successive ontogenetic growth stages. Note H-O show formation of the new central lumen, which disappears on the P section. Scale bar 2 mm.

Three specimens were serially ground and/or cut to obtain successive acetate peels and thin sections to study the earliest development of the skeleton (Figs 5 and 6).

In all transversally sectioned specimens the basal parts of the skeletons show the hollow circular central core, filled with sediment and/or calcitic cement surrounded by partly separated heterothecal and protoheterothecal layers (Figs 5C, 5D, 5K, 6B and 6C). The next sections show the central core crossed by a convex tabula, confirming that they have cut the space above the basal (transverse) tabula (Figs 5E, 5L and 6D). The diameter of the central core is constant, whereas that of the heterotheca decreases continually upward. In the next sections, the first four parasepta that developed on the tabula within the central core are centrally united by an oblique (central) paraseptum (Figs 5F, 5M, 6E and 6F). This stage of growth is continued upward and new parasepta are created by dichotomous division of the existing four parasepta (Figs 5G, 5H, 5N–5P and 6G–6N).

## The branching process

The formation of a new branches of *Oligophylloides* in the study material is restricted mostly to the later stages of the colony growth, but in two juvenile specimens it appeared relatively early in the colony development (Figs 2C and 6).

### Early branching

Owing to the scarcity of juvenile specimens, only one was cut to prepare thin serial transversal sections (Fig 6), while a second one (Fig 2C and 2D) was investigated using x-ray microtomography. Unfortunately, due to the sparite infilling of the specimen, scanning resulted in very low attenuation differences between the skeleton and the filling [24]. Consequently, these CT-scans do not show sufficient contrast to precisely trace the differences in internal morphological characters and hence detailed analysis of ontogeny using this method is only used to support observations from sections and peels. The serial sections show the successive stages of the early growth of the stem with the internal structure of the parent and, in a thickened part of the stem, the early stages of a daughter branch (Fig 6).

The central core of the daughter branch can be traced in a number of distal sections (Fig 6F–6N) before it disappears (Fig 6O and 6P), perhaps indicating colony death before the branch could develop further. The remainder of the colony is unbranched. Successive stages of the earliest ontogeny in this specimen display similar development to specimens representing unbranched stems. The difference in the axis is seen at the level of the beginning of the branching process, where the central core of the daughter branch, complete with its paraseptal apparatus, can be seen to form in the existing protoheterothecal layer of the stem. The new central core and paraseptal apparatus appear within the crescentic loculus formed within the protoheterotheca (Fig 6F–6I). Hence, there is no connection between the central cores of parent and daughter branches and the parasepta are not inherited from the parent to the daughter branch. The central core of the daughter branch, with its parasepta, initially grew at an angle to the central core of the parent, but in later growth it gradually became parallel and circular in its diameter. Both central cores are separated by protoheterothecal layers and their maximum distance apart is about 2–3 mm (Fig 6J). The paraseptal apparatuses of both central cores are composed of three generations of incomplete, dichotomously dividing parasepta. In subsequent levels the central core of the daughter branch comes closer to that of the parent and gradually decreases its diameter until it gradually disappears (Fig 6J–6N), fully covered by the protoheterothecal layers of the parent branch. In the stem above this level, only the central core of the parent branch is present and all successive growth is unbranched (Fig 6M–6P).

### Late branching and anastomosis of the branches

**Fig 7:**
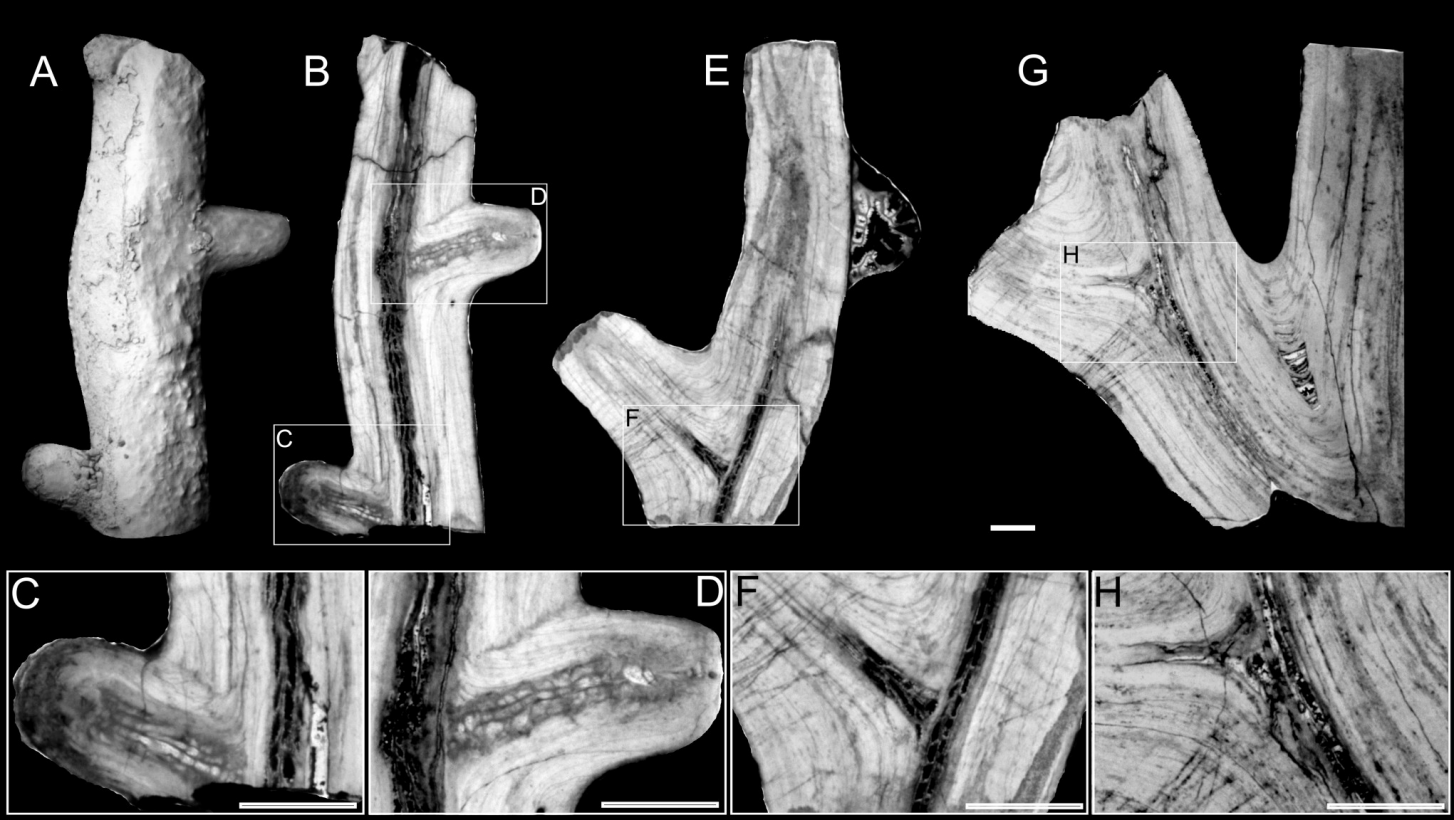
Broken fragments of colonies of *Oligophylloides* sp. showing the branching process. A-D–specimen UAM/He/B/025; E, F–specimen UAM/He/B/026; G, H–specimen UAM/He/B/027. A–external view; B–longitudinal thin section through the central lumen; C, D–enlarged fragments of the thin section (B) showing branching points; E–longitudinal thin section through the central lumen; F–enlarged fragment of the thin section (E) showing branching point; G–longitudinal thin section through the central lumen; H–enlarged fragment of the thin section (G) showing branching point. Scale bars 2 mm.

**Fig 8:**
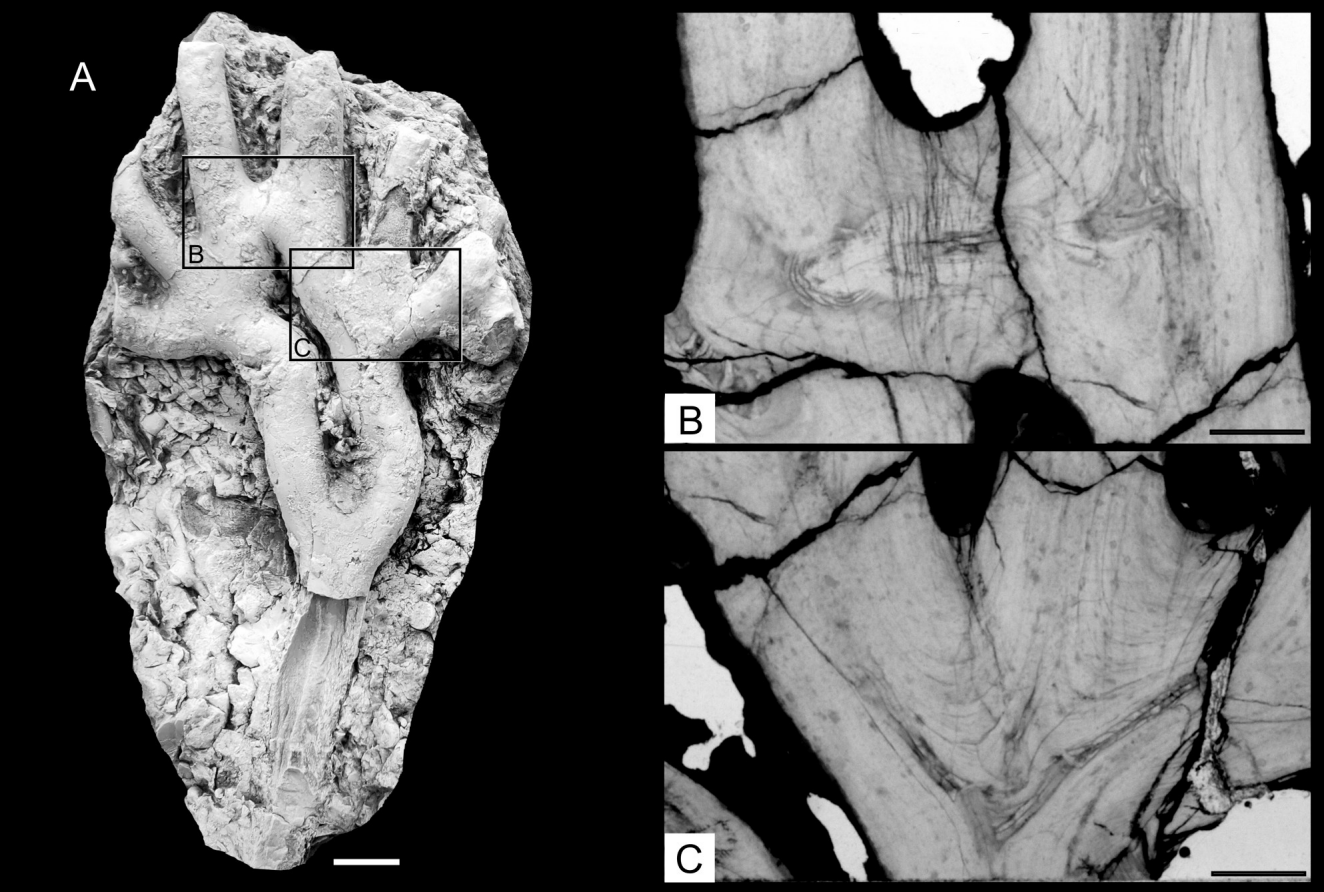
A—Fragmentary preserved fan shaped colony of *Oligophylloides* sp.–specimen UAM/He/B/030. Note: Reconnection of two neighbouring branches is well visible. Scale bar 5 mm; B–thin section of the fragment showing internal structure of the reconnection of the branches; C–thin section of the point of divergence of the branches. Scale bars for B and C 2 mm.

Fragmentarily preserved colonies of *Oligophylloides* composed of at least two branches are relatively common in the studied material. The largest branching specimen is 270 mm long. The notable character of these larger examples is that they all developed their branches in one plane, forming more or less fan-shaped colonies (Fig 8A). The branching points in some of the specimens were investigated in longitudinal thin sections (Figs 7 and 8) and the following characteristics of the branching process have been observed: 1) the formation of a new (daughter) branch always begins within the existing protoheterothecal layer of the parent branch; 2) the thickness of the protoheterotheca at the point of divergence may vary markedly depending on the growth stage of the parent branch: it can be very thin, appearing almost absent (Fig 7H), or thicker with both branches clearly separating (Figs 7C–7E and 8C).

In any case, the central core of the daughter branch is not connected to the parent’s branch central core and paraseptal systems created in both cores developed independently.

The distal parts of the branches are mostly broken in the studied material, but one specimen with two, short branches with intact growing tips (Fig 7A and 7B) may represent a more distal part of the colony. However, as their central cores are terminally covered by thick layers of protoheterotheca, they most plausibly represent branches which did not develop further.

Anastomosing branches are present in one specimen (Fig 8), in which two neighbouring branches are joined together for a short distance. The thin section shows that the two neighbouring branches are connected by the daughter branch of the right parental branch, which merged with the left neighbouring branch (Fig 8B). The internal structure shows that the central core continues into the wall of the left branch where it is surrounded by protoheterothecal layers (Fig 8B).

## Discussion

This study of *Oligophylloides*, along with former investigations of heterocorals, shows that new interpretations of their anatomy, and hence their origin and possible affinities, should be considered. Based on the skeleton structure (especially the holdfast, central core, heterotheca and protoheterotheca) and branching patterns, it seems most probable that Heterocorallia are exclusively colonial forms, developing their skeleton in a manner strikingly similar to some groups of recent gorgonian octocorals of the family Isididae (see discussion below).

### Recruitment and the development of the basal skeleton

Those coral specimens investigated here which represent early ontogenetic growth stages show unique development of skeletal elements unknown in any other Palaeozoic coral groups. Little is known about post-larval development in octocorals, but it is commonly accepted that extant octocoral planulae undergo abrupt metamorphosis after their recruitment and settlement [25] and basal skeletal elements are formed immediately after the settlement. Initial colony growth may involve either a single planula, as in *Alcyonium* [26] or single or multiple planulae, as in *Corallium* [27]: the latter case resulting from a fusion of two planulae. In recent septate corals (scleractinians) metamorphosis is followed by the formation of a basal disc and the first protosepta within the mesenteries once the gastrovascular cavity of the founder polyp has formed [28]. This critical point of the early development is unknown in the fossil record. Consequently, observations of the early ontogenetic stages in Palaeozoic corals (mainly rugosans) are restricted to basal skeletal elements, i.e. the basal disc and primary septa surrounded by the epitheca [e.g. 8,9,29]. The analysis of the external and internal morphology in juvenile forms of the heterocoral *Oligophylloides* reveals that the first skeletal element secreted by the colony was probably the holdfast, as it is the case in modern octocorals. After settlement the planula larva underwent metamorphosis to a feeding polyp that produced a membranous basal coenenchymal expansion, possibly budding more polyps in the process in a manner such as that illustrated by Costantini et al. (2018, Fig 2C) [27] for *Corallium*. This coenenchyme laid down a calcareous basal disc leading to the production of the holdfast. The initial development incorporated the secretion of the proximal part of the tubular axis–the stem–and the creation of the first tabula, which may have been covered by soft tissues. After that the process within the central core was repeated and the second arched tabula was constructed, which served as the basis for parasepta surrounded by heterothecal layers. A similar stage was also figured by Weyer (1997, Fig 2) [5], who suggested, however, that this type of the first stages lacking ’septa’ is rather anomalous and unique. The material investigated here (more than 20 juvenile specimens) shows that this mode of development of early skeleton is typical and the first parasepta appeared above the second convex tabula.

An approximately discoid holdfast is present in all of the well-preserved juvenile specimens studied. Their internal structure, studied in transverse and longitudinal sections, displays cavities within the heterothecal and protoheterothecal layers yet they form a solid basis for the development of the stem and daughter branches. Two types of cavities have been observed. The first, and most common, are relatively wide, empty and crescent-shaped, located between relatively thick sets of protoheterothecal layers in the holdfast (Figs 4D, 4E, 5D–5F and 6B–6E). It cannot be determined if these cavities are formed by separation of laminae or have developed as normal loculi. The second, found only in the holdfast and basal stem of one small specimen (Fig 5K–5N), are small and bulbous in shape. Structures with a strikingly similar appearance to the latter can be found in the axes of modern gorgonians but these are restricted to the Holaxonia and the calcaxonian Primnoidae, and consist of aggregates of fibrous calcareous material or gorgonin fibres within the predominantly proteinaceous axis [30,31]. Noé et al. (2007) found similar structures in a fossil primnoid [32].

In the majority of modern gorgonian genera, the skeletal axis is constructed from successive, more-or-less concentric lamellae or growth rings, as it is the case in *Oligophylloides*. These axes can be entirely proteinaceous, mostly proteinaceous with various amounts of calcareous material (magnesium calcite), or mostly calcareous with various amounts of proteinaceous material [30,33,34]. In the genus *Primnoa* Lamouroux, 1812, fibrous calcareous material is interspersed in the gorgonin matrix [35], while in the so called “bamboo corals” (family Isididae), despite the solid carbonate appearance of the internodes, gorgonin is present as fibres or sheaths interspersed throughout the calcareous matrix and associated with calcite crystal bundles [36,37]. Primnoidae and Isididae, especially Primnoinae and Keratoisidinae, are closely related, as indicated by molecular studies [38,39], and a transverse section of the axis of one appears rather like the negative image of a section of the other, see e.g. Williams et al. (2017, Fig 1) [35] and Noé & Dullo (2006, Fig 5) [37]. We therefore postulate that the loculi in *Oligophylloides* may be interpreted as cavities originally filled with an organic phase and that homologies exists between the wall (proheterotheca and heterotheca) of heterocorals and the stem of certain gorgonians. Both are part of a wide, basal attachment structure, which serves to anchor the relatively long colony stem to the substrate. These characteristic basal parts of heterocorals are strikingly different from those of the basal parts of all septate corals described to date. Moreover, [6] and [17] described more advanced root-like holdfasts, which were named paracolonies. Such big basal parts could be formed as an effect of multiple larval settlement and/or formation of additional branches on the root-like holdfast.

### Coloniality—corallites or branches?

Weakly dendroid colonies have been observed in several taxa of heterocorals, i.e. *Oligophylloides* [see studies by 1,3,5–7], *Hexaphylia* [40] and *Radiciphylia* [41]. The ’budding process’ of previous authors (referred here to as the formation of new branches of the colony) in heterocorals, especially in *Oligophylloides*, was studied in detail by [3] and [6]. Both these studies, together with the observations presented in this paper, show that the new (daughter) branches always begin in the existing protoheterotheca and their central core is never connected to the central core of the parent branch. The distance separating both central cores at the initial stage of the formation of the daughter branch may vary markedly depending on the number of heterothecal lamellae laid down in the parent axis. Therefore, since daughter branches could be formed at different stages of the parental growth, when the separating heterotheca is thin, the branching must have appeared relatively early, and when it is thick, it must have occurred in a late growth stage of the parental branch. In any case, the paraseptal structure located in the central core of the parent branch is not connected to the parasepta formed in the central core of the daughter. This phenomenon is not known in any other septate coral groups, where the new offsets always partly inherit the septal structure of the parent corallite. As a result, the previously postulated homology between true septa in hexa- and rugose corals on one hand, and *Oligophylloides* on the other, can be rejected. The anastomoses of the branches, as figured in this article, would be a character hard to explain if each branch represented one corallite.

It is commonly accepted that heterocoral skeletons were presumably covered by soft tissue and that the budding process (formation of the new branches) took place within this tissue. Moreover, all previous studies suggested that each branch of the skeleton represented one corallite (skeleton of single polyp). This view was supported by the conviction that the ’septal’ apparatus present in central cores of heterocorals is homologous to the septal apparatus of single corallites in other septate corals e.g. rugosans or scleractinians, being formed within the mesenteries of a single polyp. What is more, as described above, the protoheterotheca and heterotheca are composed of continuous layers covering precedent ones, thus evidencing continuous skeleton coverage by soft tissues.

In the light of the analyses of the *Oligophylloides* skeletons presented here, we hypothesise that the stem and branches of heterocorals might, in fact, represent the skeleton of a multi-polyp colonial coral. Given the considerable depths of environments in which *Oligophylloides* usually occurred, at the lowermost photic or dysphotic zone [e.g. 15], photosymbiotic mode of life seems unlikely and therefore we assume its heterotrophic mode of life, similar to that of recent deep-water gorgonians [42]. Assuming that these corals were heterotrophic, it seems highly implausible that a single polyp of potential diameter less than 10 mm located at the top of a branch could cover the energetic budget of the entire volume of living tissue, which covered the entire skeleton. Conversely, if we accept a similar colony structure as the one present, for example, in Isididae, we can assume that only multiple feeding polyps in a colonial animal could cover the energetic needs of the colony. In this case, the ’septal apparatus’ and ’tabulae’ inside the central core form a part of coenosteum and were not elements of a single corallite, as postulated by previous authors since the erection of the order Heterocorallia by Schindewolf in 1941 [2]. As outlined before, earlier studies of the ’septal apparatus’ in heterocorals showed that their arrangement may completely change or that some of them may disappear or temporarily reappear during ontogeny [1,3,4], which, conceivably, would anatomically be impossible for a single polyp. Thus, it is suggested here that the tabulae and parasepta in the central core were secreted by the common tissue of the growing tip or by a specialized part of the soft body.

### Comparisons with Octocorallia

The skeletons of *Oligophylloides* show a number of similarities with octocorals. First of all, the mineralogy of modern octocorals is strikingly similar to that of *Oligophylloides*. Octocoral sclerites, as well as axial skeletons, are composed of calcite and/or high-magnesium calcite [43]. Secondly, the overall arborescent shape, with branching in one plane (fan-shaped colony) is common in many octocorals, such as genera within the Plexauridae Lamouroux, 1812 (Holaxonia), the Coralliidae Lamouroux, 1812 (Scleraxonia) and notably in the Isididae Lamouroux, 1812 (Calcaxonia). Noé & Dullo (2006, Fig 10) [37] and [32] have shown that the growth pattern of *Keratoisis* Wright, 1869, where the youngest parts of the skeleton are composed of a smaller number of skeletal layers, and this character is the same as in *Oligophylloides*. In many extant octocorals, gorgonin fibres are mineralized–this feature gives them resistance to torsion [44]. Genera within the family Isididae, for example, possess organic nodes which alternate with highly calcified internodes (magnesium calcite). Organic nodes result in high flexibility of the whole skeleton, but most gorgonians do not possess them and their axial skeletons are continuous. And, as previously mentioned, in the majority of genera, their axes display concentric rings similar to those of *Oligophylloides*.

**Fig 9:**
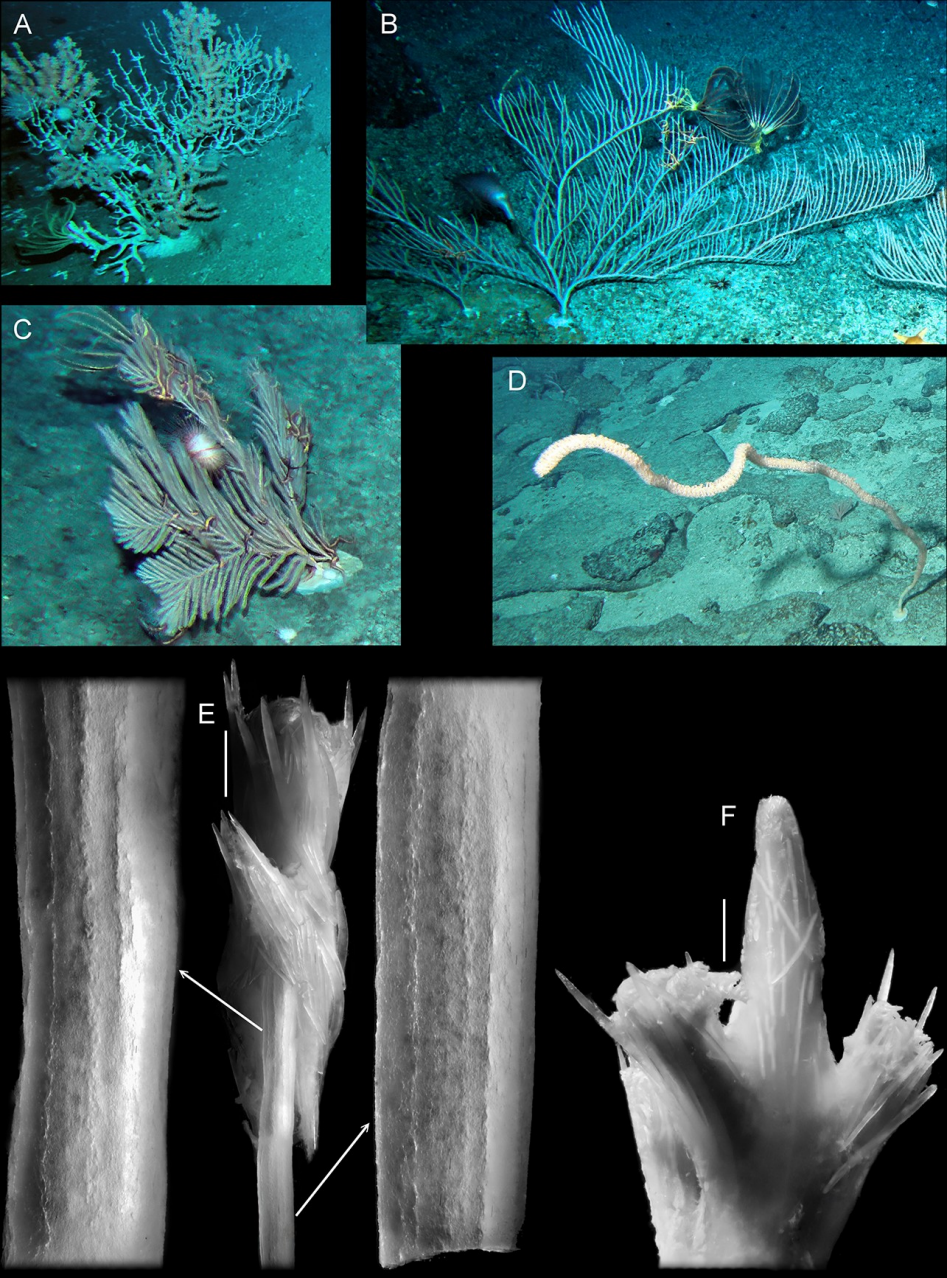
A-D, Underwater images of colonies of some Calcaxonia with a holdfast. A–*Keratoisis* sp.; B–*Paracalyptrophora* sp.; C–*Callogorgia* sp.; D–*Lepidisis* sp.: E–branch tip of *Isidella* sp. b with a terminal polyp and close-ups of axial internodes; F–branch tip of *Isidella* sp. a without terminal polyp. Scale bars 1 mm E-F.

The structure of the ’wall’ (heterotheca and protoheterotheca) strongly resembles that of a number of octocorals. Representatives of *Primnoa* (Primnoidae, Calcaxonia) show similar separations of laminae of the more calcified skeletal layers [31]. Although these might be due to dehydration [45], similar structures can be seen in the holdfast of a specimen of *Ellisella* Gray, 1858 illustrated by Grasshoff et al. (1983, pl. 1, Fig 20) [46]. The vesicle-shaped loculi visible in *Oligophylloides* (Fig 5K–5N) also occur, for example, in *Plexaurella vermiculata* (Lamarck, 1816) illustrated by Kükenthal (1919, pl. 51, Figs 101 and 102) [31]. Other types of loculi similar to that shown on the Fig 5E, from both holaxonians and calcaxonians, are illustrated in Kükenthal (1919, Figs 100–118) [31]. By definition, a hollow axial skeleton occurs in all holaxonian octocorals and also in a number of genera within the calcaxonian subfamily Keratosidinae (family Isididae) [e.g. 31,34,37,47–49]. Longitudinal ribs and furrows are visible on the surface of the axial skeletons of many octocorals [e.g. 48,49], and these crenulations (Fig 9A–9C) correspond to the position of longitudinal gastrodermal canals within the soft tissue [48]. Such longitudinal crenulations, although rarely preserved, are also present in *Oligophylloides*, see: e.g. Chwieduk (2001, pl. 1, Fig 2) [3], Berkowski (2002, pl. 17, Figs 1 and 2), [13] and here above in Figs 3 and 4A.

The present study demonstrated that discoid or subdiscoid holdfasts are typical for *Oligophylloides*. Similar types of discoid holdfasts, calcareous and/or proteinaceous, are known in many gorgonian octocorals [e.g. 47]. In rare cases, representatives of *Oligophylloides* also formed complex holdfasts, such as those shown by Weyer (2017, pl. 10, Fig 16) [6] and [17]. Holdfasts showing the same complex structure are known from extant and subfossil representatives of the genus *Keratoisis* [32]. Asymmetric growth seen in the latter genus is most probably a result of overgrowing an uneven substrate [32], a similar case seen on Weyer’s material, where the coral overgrew a crinoid skeleton [6].

Representatives of *Oligophylloides* possess a hollow central core containing structures referred to as ’septa’ in previous publications [e.g. 1,3,4,6,13]. Although no structure of this kind is known from modern octocorals, genera in the calcaxonian family Isididae have a solid, mostly calcareous, axis formed from concentric growth-rings and some in the subfamily Keratoisidinae, such as *Keratoisis* Wright, 1869, *Lepidisis* Verrill, 1883, *Isidella* Gray, 1858 and *Jasonisis* Alderslade & McFadden, 2012, also have a hollow central core (at last in the younger parts of the colonies) within which tabula-like structures [49,50] are present, giving the axes in these taxa some distinct similarities to that in *Oligophylloides*. To investigate axial similarities further, we examined samples from several genera, focussing primarily on the growing tips of branches, in order to compare them to the ’distal cone’ in our *Oligophylloides* material. The developing tip of the axis was found to be extremely thin and fragile, and due to the rigours of deep-water collection by beam trawl they tend to be crushed. Several, however, were recovered mostly or completely intact.

**Fig 10:**
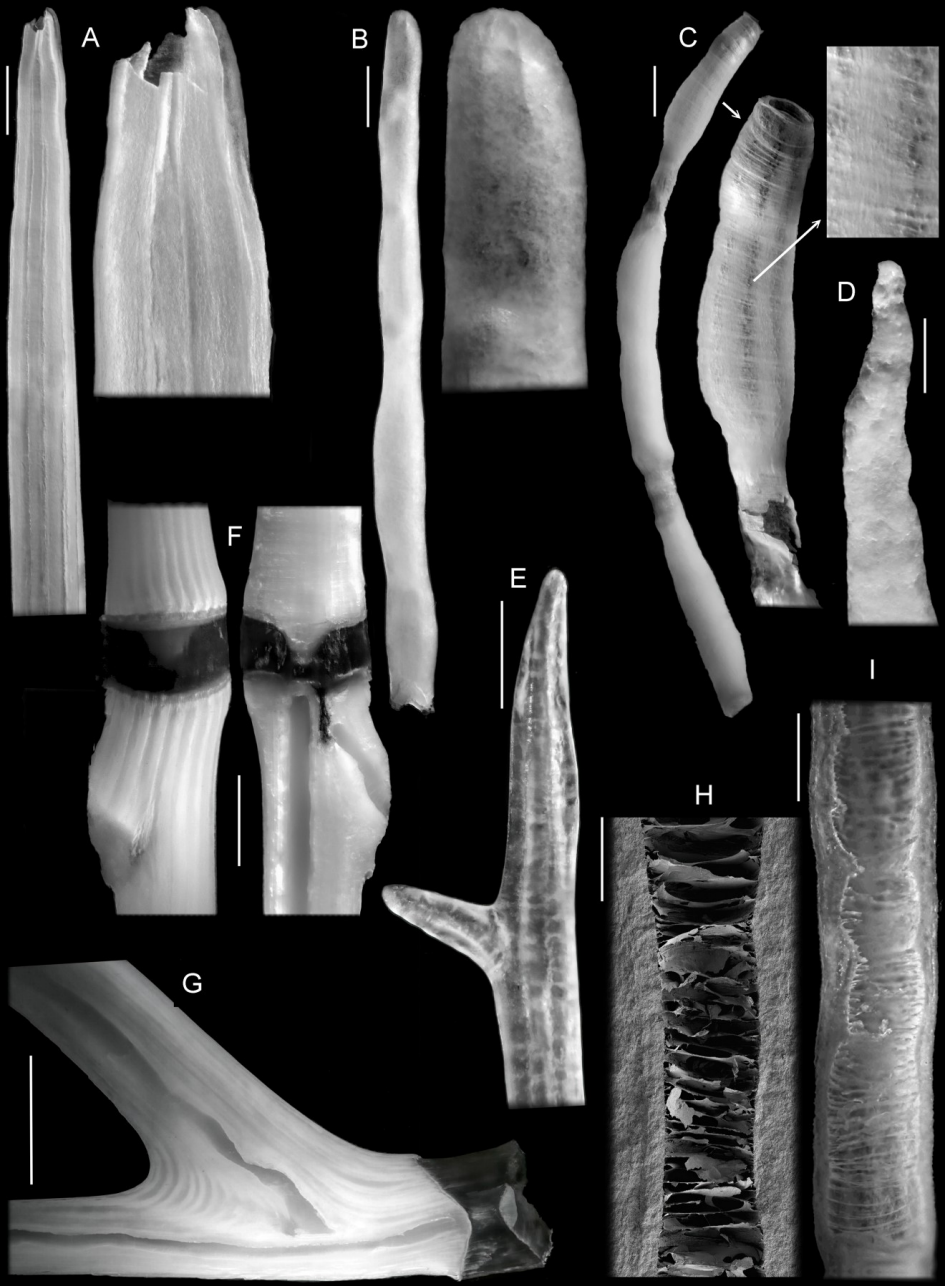
A-E, Terminal axis portions of colonies of Isididae, some with a close-up of the tip. A–*Isidella* sp. a; B–*Keratoisis* sp. a; C–*Jasonisis thresheri*; D–*Acanella* sp. d; E–*Primnoisis* sp. n; F–exterior and interior aspects of a portion of a *Keratoisis* sp. k axis showing non-confluent central core; G–interior aspect of a portion of a *Keratoisis* sp. k axis showing non-confluent central core; H–detail of interior of hollow core of *Jasonisis thresheri* axis–SEM image; I–detail of interior of hollow core of *Jasonisis thresheri* axis–light microscope image. (H and I previously published in *Zootaxa*, 2012). Scale bars 1mm A-C, G, I; 0.5mm, F; 0.2 mm D.

In some colonies, branch tips were found to have a terminal polyp (Fig 9E) while in others, there was no terminal polyp and the tip was tapered and rounded, but the coenenchyme was reinforced with numerous needle-like sclerites (Fig 9F). In all instances where longitudinal, external ridges were present on the developing axial internodes, they were most pronounced in the distal part, diminishing or becoming completely absent on those immediately proximal: for example, the arrowed internode close-ups in Fig 9E. Unfortunately, the actual internode tip in this specimen of *Isidella* was not intact, however, that in the *Isidella* sample illustrated in Fig 9F was almost complete and is depicted in Fig 10A. As can be seen in the close-up of the most distal portion, the youngest parts of the external ridges are flange-like and extremely well developed compared to those in the older parts of the internode. Noé & Dullo (2006) [37] reported the growing tip of branch buds to have the central core of the axis terminally open in a colony of the closely related genus *Keratoisis*. Fig 10B represents the developing axis from a colony of *Keratoisis*. As can be seen in the close-up of the terminal region, external ridges, although hardly pronounced, are only present at the very tip. In thicker older parts of this colony, longitudinal ridges are very visible.

Three internodes, separated by internally developing nodes, from the tip of a branch of *Jasonis thresheri* Alderslade & McFadden, 2012 are illustrated in Fig 10C, together with a close-up of the most distal internode and detail of its surface. On the most distal internode, longitudinal ridges were forming from clumps of granules and are difficult to make out. They are more continuous on the preceding internode, but cannot be detected on the one before that. In thicker, older parts of this colony, however, longitudinal ridges are easily visible. The axis in this species has the most developed central core of all known genera in the Keratoisidinae, having delicate, complete, densely arranged tabula-like partitions (Fig 10H and 10I) and fine cobweb-like structures spanning the cavity [49]. The developing terminal node is quite different to those detailed above. Not only is it somewhat bulbous and sausage-shaped, and lined internally with fine, flange-like, circumferential rings that are visible through the thin, glassy wall, but it appears that, during development, the extremity of the central core is open. Two similar examples were examined and in neither was there any evidence of fractured material that could have been a cap (this colony is in good condition, having been collected by an underwater Remotely Operated Vehicle).

It should also be noted that mineralised tabula-like structures can also occur in the central core of the proteinaceous axis of holaxonian genera and, although discrete parasepta are not formed, the chambers between these dissepiments may contain mineralised fibres as illustrated by Bayer & Macintyre (2001, Figs 4, 8 and 9) [30].

A couple of examples of axial development in isidid genera that do not have a hollow central core were also examined: *Acanella* Gray, 1870 and *Primnoisis* Studer & (Wright), 1887. The former tends to have rather weak, external longitudinal axial ridges and the developing tip of the axis had a very rough surface and no sign of ridges (Fig 10D). *Primnoisis* does have a ridged axis and ridges maybe present at the tip of a developing internode (Fig 10E).

The central core in the axis of a daughter branch in *Oligophylloides* begins within the axis of parent branch close to the central core but is not confluent with it. The same characteristic was reported for *Keratoisis* by Noé & Dullo (2006, Fig 3c and 3d) [37] and we were able to confirm this in samples of two different species of that genus (Fig 10F and 10G). Apart from using nodal versus internodal branching to distinguish between the very similar genera *Isidella* and *Keratoisis*, differences in detailed axial characteristics have not been comprehensively evaluated. However, a similar type of formation of new branches was recorded in some recent isidid gorgonians by Noé & Dullo (2006, Fig 3C and 3D) [37], where the hollow central core of the daughter branch begins close to the central core in the axis of the parent branch.

An important, although not always present, character of most octocorals is the presence of sclerites within the coenechyme of the colony. In the studied material of *Oligophylloides* and other previously investigated heterocorals, the sclerites have not been identified. They are absent within the skeleton and no traces of this type of structures have been observed in the sediment surrounding heterocoral skeletons in thin sections. The lack of sclerites may be interpreted in two ways: first, they were not present within the soft body of the colony, similarly as in some octocorals that do not possess sclerites, for example representatives of the soft-bodied coral genera *Altumia* Benayahu, 2017 and *Hadaka* Lau & Reimer, 2019, or the gorgonian genera *Huziogorgia* López-González, 2020 and *Acanthoaxis* Ofwegen & McFadden, 2009. A second possibility is that primarily aragonitic sclerites were not preserved. Extant octocorals may possess calcitic and aragonitic elements within one colony [30] and it is possible that primarily calcitic *Oligophylloides* could have had aragonitic sclerites.

The majority of modern gorgonian octocorals possess axial skeletons composed of mineral and organic phases, and the individuals expose a maximum area to the current [51]. Within Isididae, all corals have (predominantly) organic nodes and calcareous internodes, that both interplay in giving the skeleton the required stiffness or elasticity in the given hydrodynamic regime, to which the mechanical properties of the axis adjust. Thus, differential stiffness may be a result of occupying different ecological niches [52]. Corals living in low-energy environments may not need very tensile skeletons and composite organic-mineral skeletons devoid of organic nodes might have been sufficient in low hydrodynamic regime near the deep seafloor [52]. Due to their similar skeletal characteristics, members of the Isididae are worth considering as an ecological equivalent of *Oligophylloides* representatives.

The fossil record of octocorals is scattered, but concentrated in the lower Palaeozoic. Some Cambrian fossils were assigned as representatives of octocorals (e.g., *Pywackia*), but the arguments in favour of their octocoral affinities are disputable and no convincing Cambrian octocoral has been described so far [53–56]. Ordovician, Silurian and Carboniferous rocks have yielded some less questionable octocoral fossils [57–59]. Fernández-Martínez et al. 2019 [60] described unequivocal octocoral sclerites associated with a tabulate coral. Octocorals as a group are monophyletic [39,61–63] and their higher-level molecular phylogeny is relatively well known. Some smaller taxonomic groups may, however, be poly- or paraphyletic, as is in the case of *Keratoisidinae* [64]. The most recent fossil-calibrated phylogenetic analyses [65] placed the divergence of octocorals most probably in the Ediacaran (95% posterior density estimate 578 Ma, range 685–483 Ma, that is Cryogenian–Ordovician). Therefore, molecular data combined with the fragmentary fossil record point to the origin of octocorals between the late Precambrian and the early Palaeozoic. Quatrini et al. (2020) [65] also showed that the appearance of the high magnesium calcite axial skeletons of octocorals took place *c*. 438 Ma (516–362 Ma, Cambrian-Devonian). In the light of these data, the appearance of calcareous skeletons of octocorals in the Devonian seas is consistent with known fossil record of Octocorallia and gives support to our reinterpretation of *Oligophylloides*.

We can therefore conclude that all, or nearly all, structures of *Oligophylloides* coralla can be interpreted as analogous to similar structures occurring in octocorals. Given their similar spatial arrangement and morphology is convergent with many recent octocorals, we finally arrive to the conclusion that they are homologous, and this genus should be assigned within octocorals.

## Conclusions

A new concept of the order Heterocorallia is presented basing on the detailed analyses of the skeleton of juvenile and branching specimens of the genus *Oligophylloides* and comparisons with extant octocorals.

**Fig 11:**
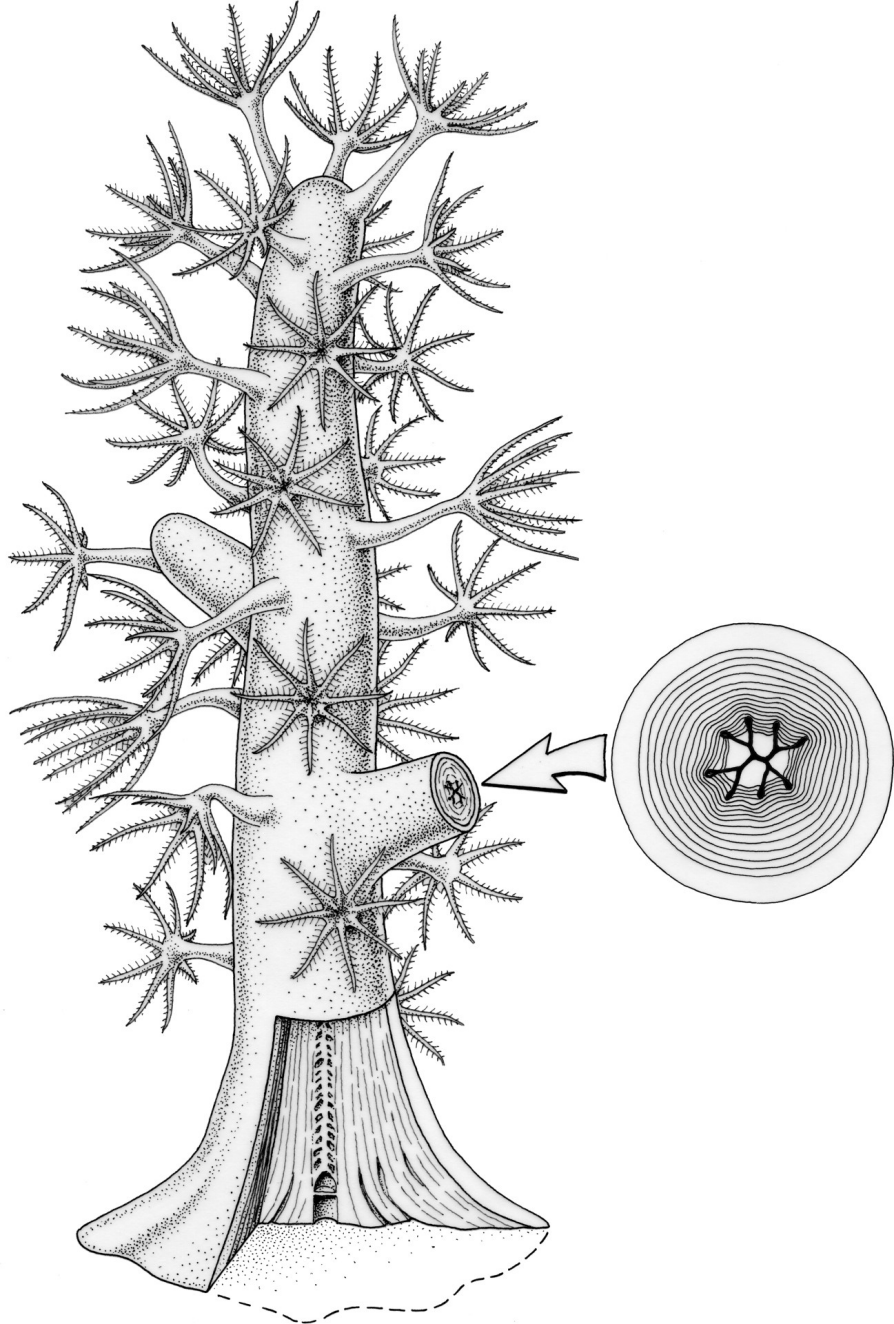
Reconstruction of the juvenile colony of *Oligophylloides*. Drawing by B. Waksmundzki.

1. The extinct order Heterocorallia was represented strictly by colonial forms (see: reconstruction of presumed juvenile colony on Fig 11). The axis supported a coenenchyme layer with multiple individual polyps and each branch formed part of the common corallum and not of an extended corallite.
2. The early development of the skeleton of *Oligophylloides*, and presumably other heterocorals started from a discoid holdfast created by the soft tissue, which formed thick heterothecal and protoheterothecal layers extending laterally.
3. Within the holdfast, the proximal portion of the hollow cylindrical stem was laid down with an aseptate tabula surrounded by heterothecal layers and the first paraseptal structure formed on the second elevated tabula.
4. The growing tip of heterocorals was figured and studied for the first time and show that the development of paraseptal apparatus (dichotomously dividing parasepta) took place not only in the central lumen, but also within heterothecal layers. It also shows that the tips did not have corallites or corallite-like structures.
5. The branching process started in the protoheterothecal layers, so the central core of the parent and daughter branch were not connected.
6. We demonstrated that many skeletal structures of *Oligophylloides* (holdfast, wall composed of protohetero- and heterotheca, parasepta and growing tip) can be interpreted as homologous with similar skeletal structures in octocorals, most notably representatives of Keratoisidinae. We propose including *Oligophylloides* within the subclass Octocorallia Haeckel, 1866. Such inclusion is here only provisionally referred to other heterocorals, excluding Palaeozoic caliculate corals revealing heterocoralloid symmetry of septal apparatus, known as Calyxcorallia [4]. This, however, requires further studies of type specimens of other typical heterocorals, especially *Heterophyllia* and *Hexaphyllia*. Our taxonomic interpretation is in agreement with the known stratigraphic range of the octocoral fossil record and their divergence time.

## Systematic palaeontology

                    Subclass OCTOCORALLIA Haeckel, 1866

                 Order HETEROCORALLIA Schindewolf, 1941

*Genera assigned*. *Heterophyllia* McCoy, 1849; *Hexaphyllia* Stuckenberg, 1904; *Mariaephyllia* Fedorowski, 1991; *Oligophylloides* Różkowska, 1969, *Radiciphyllia* Sugiyama, 1984; *Stellaphyllia* Fernández-Martínez, Tourneur & López-Alcántara, 2003

*Emended diagnosis*. Octocorals possessing coralla forming solitary or branching stems. Central core with parasepta dichotomously dividing adaxially from the central primary paraseptum and forming generations of parasepta. Additional tabulae-like structures may be present in central core. Wall surrounding central core composed of compact calcite layers of a heterotheca or protoheterotheca type.

*Stratigraphic distribution*. Middle Devonian–Carboniferous.

                 Genus *Oligophylloides*, Różkowska, 1969

*Type species*. *Oligophylloides pachythecus* Różkowska, 1969; from the Famennian of Holy Cross Mountains, Poland.

*Emended Diagnosis*. Heterocorals with colonies composed of a solitary or branching stem forming fan-shaped coralla. Holdfast discoid in youth, in later growth may form large root-like complex structures. Distal growing tip with furrows and ridges. Wall is very thick, of heterotheca-type close to the central core covered externally by smooth thick layers of protoheterotheca. Central core with paraseptal apparatus developed in heterocoralloid manner and tabulae-like structures.

*Stratigraphic distribution*. Upper Devonian, upper Famennian.

                 *Oligophylloides* sp.

                     Figs 2–8

*Material*. 26 small specimens (*c*. 15–36 mm long), representing juvenile (proximal) parts with holdfasts, and more than 30 broken, fragmentary branched sections representing more distal parts of the coralla. Figured specimens: UAM/He/B/011-030.

*Stratigraphic and geographic distribution*. Upper Famennian. Jebel Bou Ifaherioun, Anti-Atlas, Morocco.
